# Optimization of simultaneous screening of the main mutations involved in non-syndromic deafness using the TaqMan® OpenArray™ Genotyping Platform

**DOI:** 10.1186/1471-2350-14-112

**Published:** 2013-10-24

**Authors:** Fábio Tadeu Arrojo Martins, Priscila Zonzini Ramos, Maria Carolina Costa Melo Svidnicki, Arthur Menino Castilho, Edi Lúcia Sartorato

**Affiliations:** 1 Human Molecular Genetics Laboratory, Center for Molecular Biology and Genetic Engineering (CBMEG), State University of Campinas – UNICAMP, Campinas, SP, Brazil; 2 Department of Otorhinolaryngology, Head and Neck, Faculty of Medical Sciences, State University of Campinas – UNICAMP, Campinas, SP, Brazil

**Keywords:** Genotyping, OpenArray™, High-throughput, Deafness, Genetics, Hearing loss

## Abstract

**Background:**

Hearing loss is the most common sensory deficit in humans, affecting approximately 10% of the global population. In developed countries, one in every 500 individuals suffers from severe to profound bilateral sensorineural hearing loss. For those up to 5 years old, the proportion is higher, at 2.7 in 1000 individuals, and for adolescents the average is 3.5 in 1000. Among the causes of hearing loss, more than 50% are related to genetic factors. To date, nearly 150 loci and 64 genes have been associated with hearing loss. Mutations in the *GJB2* gene, which encodes connexin 26, constitute the main genetic cause. So far, more than 300 variations have been described in this gene.

As a response to the clinical and genetic heterogeneity of hearing loss and the importance of correct molecular diagnosis of individuals with hereditary hearing loss, this study worked in the optimization for a diagnostic protocol employing a high-throughput genotyping technology.

**Methods:**

For this work, was used the TaqMan® OpenArray™ Genotyping platform. This is a high performance, high-throughput technology based on real-time PCR, which enables the evaluation of up to 3072 SNPs (Single Nucleotide Polymorphisms), point mutations, small deletions, and insertions, using a single genotyping plate. For the study, were selected the layout allowing to analyze 32 alterations in 96 individuals simultaneously. In the end, the generated results were validated by conventional techniques, as direct sequencing, Multiplex PCR and *RFLP-PCR*.

**Results:**

A total of 376 individuals were analyzed, of which 94 were healthy controls, totaling 4 plates in duplicate. All 31 of the changes analyzed were present in the nuclear genes *GJB2, GJB6, CRYL1, TMC1, SLC26A4, miR-96,* and *OTOF*, and in the mitochondrial genes *MT-RNR1* and *MT-TS1*. The reactions were subsequently validated by established techniques (direct sequencing, multiplex PCR, and *RFLP-PCR*) that had previously been used to perform molecular screening of hearing loss at the Human Genetics Laboratory of the Center for Molecular Biology and Genetic Engineering (CBMEG), at the State University of Campinas (UNICAMP). In total, 11,656 genotyping reactions were performed. Of these, only 351 reactions failed, representing approximately 3.01% of the total. The average accuracy of genotyping using the OpenArray™ plates was 96.99%.

**Conclusions:**

The results demonstrated the accuracy, low cost, and good reproducibility of the technique, indicating that the TaqMan® OpenArray™ Genotyping Platform is a useful and reliable tool for application in molecular diagnostic testing of hearing loss.

## Background

Hearing loss is the most common sensory deficit in humans. It can restrict oral skills and cause changes in cognitive and psychological development. A study undertaken in 2013 showed that at least 360 million people, corresponding to more than 5% of the global population, have disabling hearing loss (loss exceeding 40 dB in adults or 30 dB in children) [1].

One in every 500 children has severe to profound congenital bilateral deafness. For children up to 5 years of age, this number rises to 2.7 per 1000 children, and for adolescents the number is 3.5 per 1000 individuals. In the case of unilateral profound deafness, the number of children affected is 3–6 in every 1000, and 2–4 out of 1000 children will be deaf before adulthood (post-lingual deafness). These deafness rates can vary according to location [2-4].

Hearing impairment can have congenital or acquired causes. Congenital hearing loss affects individuals who are born with the loss, or acquire it shortly after birth. It is caused by genetic factors such as changes in one or more genes, or in regulatory mechanisms that are involved in the proper development, structure, and function of the ear. Acquired hearing loss occurs in the auditory system due to injuries that occur during life, such as frequent exposure to high intensity sound, acoustic trauma, infections, and ototoxic drugs, amongst other causes (Table 1) [1].

**Table 1:** Main causes of hearing loss

**Causes of hearing loss**		
**Environmental**	**Prenatal**	Maternal infection (rubella, cytomegalovirus)
		Drug use during pregnancy
		Irradiation during gestation
	**Perinatal**	Severe jaundice
		Birth injury
		Anoxia
	**Postnatal**	Otitis
		Meningitis
		Measles
		Mumps
		Trauma
**Genetic**	**Syndromic**	Autosomal dominant
		Autosomal recessive
		X-Linked
		Mitochondrial inheritance
	**Non-syndromic**	Autosomal dominant
		Autosomal recessive
		X-Linked
		Y-Linked
		Mitochondrial inheritance

Source: adapted from [5].

In Brazil, 66.7% of cases of hearing loss are caused by environmental factors, while 18.5% are of unknown etiology and 14.8% are classified as being due to genetic inheritance [6].

Studies have shown that in developed countries at least 50% of all problems related to congenital deafness are hereditary, with 70% of these cases classified as non-syndromic hearing loss (NSHL), where the deafness is an isolated symptom. The remaining 30% of cases are due to syndromic hearing loss (SHL), which is associated with other symptoms or abnormalities in addition to deafness, including malformations, vision problems, renal, thyroid, cardiac dysfunctions, etc. [7-9].

Non-syndromic hearing loss is divided into different subtypes according to the inheritance pattern: autosomal dominant (DFNA, 20%), autosomal recessive (DFNB, 80%), X-linked (DFNX, <1%), Y-linked (DFNY, <1%), and mitochondrial (less than 1%) [10-13].

To date, more than 64 genes have been reported in 150 loci related to hearing loss, and it is estimated that the number of genes could reach 300, equivalent to 1% of all human genes [3,14].

More than 134 loci are associated with non-syndromic hearing loss, and the most common mutations are found in the *GJB2* gene (which encodes the connexin 26 protein), accounting for over 50% of all cases of non-syndromic hearing loss and 10-20% of all cases of pre-lingual hearing loss in developed countries [14].

Among some others main genes related to Non-Syndromic Hearing Loss are the *SLC26A4*, *OTOF* and *TMC1* genes. Alterations in the *SLC26A4* gene are responsible to make it as the second most frequent cause of ARNSHL (Autosomal Recessive Non-Syndromic Hearing Loss). For each of these genes, excluding the *GJB2* who already have knowledge of more than 300 alterations, there are at least 20 changes already reported [15,16].

As indicated above, many genes and mutations are related to hearing loss of genetic origin. There is therefore a need for in-depth investigations to diagnose and attribute mutations detected in earlier tests, enabling a better understanding of the molecular mechanisms of hearing loss in individuals with unknown etiology and a correct treatment.

Direct sequencing is widely used, but has drawbacks in that it is expensive, time-consuming, and inefficient for the sequencing of large fragments. Variations of the PCR (Polymerase Chain Reaction) technique have been tested as cheaper options for the detection of the main mutations associated with the disease under investigation. However, these techniques have also shown severe limitations in terms of their diagnostic applicability. Independent of the particular variant of the technique, PCR results are mostly individual, or generate one result per reaction (excluding the Multiplex PCR), resulting in the process becoming expensive and unfeasible due to the cost of reagents [15].

The emergence of high-throughput technologies makes it possible to perform multiple simultaneous experiments, which could help to elucidate the etiology of different cases of hearing loss. These new technologies use only small volumes of samples and reagents in the reactions, and combine high accuracy with simplicity. A fewer number of steps, together with automation using pipetting stations, decreases both the time required for development of the experimental phase and the likely errors associated with manual pipetting, allowing several tests to be performed simultaneously at low cost [17].

In view of the clinical and genetic heterogeneity of hearing loss and the importance of correct molecular diagnosis of individuals with hereditary hearing loss, this paper proposes the optimization of a diagnostic protocol that uses a high-throughput genotyping technology based on real-time PCR, known as the TaqMan® OpenArray™ Genotyping Platform (Life Technologies). To this end, a genotyping plate was developed that enabled the simultaneous analysis of 32 changes in 96 individuals, using a single plate.

## Methods

### Casuistry

The 376 samples selected had been referred to the Human Molecular Genetics Laboratory of the Center for Molecular Biology and Molecular Engineering (CBMEG) by different organizations in order to determine the etiology of the hearing loss. Of these individuals, 86 of them were used as negative and positive controls, presenting previous results for the *GJB2* gene and/or the *GJB6* gene, and for the mitochondrial mutation m.1555A > G in the *MT-RNR1* gene. A total of 94 controls with normal hearing were employed.

This project was approved by the Research and Ethics Committee of the Faculty of Medical Sciences, UNICAMP, under protocol No. 396/2006. All the volunteers who participated in this work had previously signed informed consent forms, after having received an explanation of the intended investigation.

### The TaqMan® OpenArray™ Genotyping Platform

The TaqMan® OpenArray™ Genotyping platform (Applied Biosystems, USA) is a high performance, high-throughput technology based on real-time PCR, which enables the evaluation of up to 3072 SNPs, point mutations, small deletions, and insertions, using a single genotyping plate. This platform also allows 2688 gene expression assays to be performed per plate. The platforms for genotyping and gene expression must conform to a default layout test offered by the company [18]. Based on hydrolysis of the TaqMan® probes, the TaqMan® OpenArray™ Genotyping technique requires a pair of primers common to both wild-type and the mutant sequences, as well as two different MGB (Minor Groove Binder) probes for each assay, one being a probe for the normal sequence, labeled with the VIC® fluorophore, and the other being a probe for the mutant sequence, labeled with the FAM® fluorophore.

**Figure 1:**
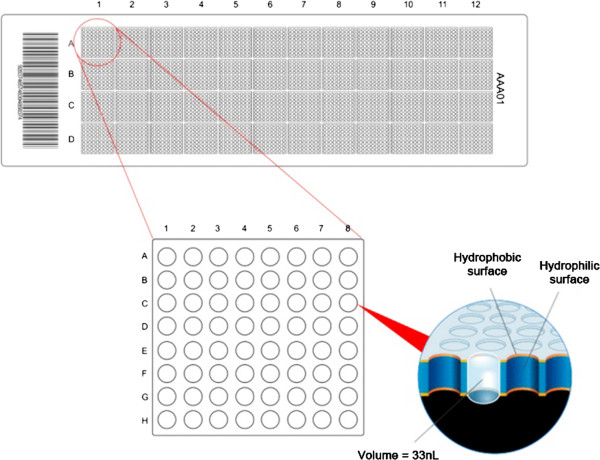
**Representation of the OpenArray™ plate.** This contains 48 sub-arrays, highlighting the arrangement of a sub-array with 64 nano-wells, each with a volume of 33 nL.

The plates used in the OpenArray™ assays were composed of 48 sub-arrays (4.5 mm × 4.5 mm), each with 64 nano-wells (Figure 1). The surface of the plate had hydrophobic properties, while the interior of the wells (where the probes and primers needed for the reactions were already installed) were hydrophilic in nature. These physical properties enabled small volumes (33 nL) of sample to be delivered with great accuracy and precision [18].

The platform consisted of a pipetting system (OpenArray™ AccuFill® System) (Applied Biosystems), connected to a computer that controlled all the operations by means of the manufacturer’s proprietary software. The platform also included a Flat Dual Block 9700 GeneAmp® PCR System thermocycler (Applied Biosystems), suitable for cycling the OpenArray™ plates, a sealing station, and an OpenArray™ NT Cycler®, which was responsible for capturing the fluorescence emitted from the plate. This last item of equipment was also controlled using the same OpenArray™ Genotyper software.

### DNA genomic extraction

The genomic DNA was extracted from leukocytes present in 10–15 mL of peripheral blood, collected in Vacutainer tubes containing 10% 0.5 M EDTA (ethylenediaminetetraacetic acid, disodium dihydrate salt) at pH 8.0. The extraction was performed using a standardized phenol-chloroform method developed at the Human Molecular Genetics Laboratory of the Center for Molecular Biology and Genetic Engineering (CBMEG), State University of Campinas (UNICAMP).

### Survey and customization of tests

After a bibliographic search for genetic alterations related to hearing loss in different populations, 32 alterations (Table 2) present in the population to be studied were selected for the development of an OpenArray™ plate containing the assays.

**Table 2:** List of 32 alterations analyzed using the TaqMan® OpenArray™ Genotyping Platform

**Gene (GenBank ID)**	**Alteration**	**Protein modification**	**Bibliographical reference**
***GJB2*** (ID 2706)	c.283G > A	p.V95M	[19]
	c.339 T > G	p.S113R	[19]
	c.279G > A	p.M93I	[20]
	c.286G > A	p.W24X	[21]
	c.439G > A	p.E147K	[15]
	c.617A > G	p.N206S	[22]
	c.385G > A	p.E129K	[22]
	c.109G > A	p.V37I	[19]
	c.269 T > C	p.L90P	[23]
	c.550C > G	p.R184W	[24]
	c.551G > C	p.R184P	[25]
	c.516G > A	p.W172X	[15]
	c.224G > A	p.R75Q	[15]
	c.101 T > C	p.M34T	[26]
	c.457G > A	p.V153I	[15]
	c.503A > G	p.K168R	[15]
	c.35delG	*Frameshift*	[27]
	c.167delT	*Frameshift*	[27]
	c.235delC	*Frameshift*	
***GJB6*** (ID 10804)	c.6013G > T		
***OTOF*** (ID 9381)	c.2485C > T	p.Q829X	[28]
***miR96*** (ID 407053)	c.13G > A		[29]
	c.14C > A		[29]
***TMC1*** (ID 117531)	c.1939 T > C	p.S647P	[30]
***CRYL1*** (ID 51084)	c.1622 T > C		
***MT-TS1*** (ID 4574)	m.7445A > G		[31]
***MT-RNR1*** (ID 6775087)	m.1555A > G		[32]
	m.827A > G		[33]
	m.1494C > T		[34]
***SLC26A4*** (ID 5172)	c.445G > A	p.G149R	[35]
	c.1238A > G	p.Q413R	[35]
	c.1826 T > G	p.V609G	[36]

The selected alterations were found in previous studies realized at the Human Molecular Genetics Laboratory of the Center for Molecular Biology and Genetic Engineering (CBMEG). Most of the selected changes were alterations related with the hearing loss, affecting the protein structure. But were selected two essays to detect the large deletions in the *GJB6* gene (rs11843171 and rs144457142). The strategy was adopted in this case because the OpenArray™ can detect insertions or deletions with only 6 bp. This strategy allowed checking if the probes would or not bind with both alleles. If there were bind in both alleles, could mean that there were not any large deletions. If any probe did not bind, so some of the deletions could be present.

The sequences (approximately 300–500 bp) covering these changes were taken from the database of the National Center for Biotechnology Information (NCBI - http://www.ncbi.nlm.nih.gov/) and Ensembl (http://www.ensembl.org/index.html). The changes were flagged, marked, and sent for customization using FileBuilder software (Applied BioSystems).

### Sample preparation

The purity and concentration of the samples were checked using a NanoDrop® 8000 spectrophotometer (Thermo Scientific) and a Qubit® 2.0 fluorometer (Invitrogen), respectively.

For the samples showing an A_260/280_ ratio between 1.7 and 2.0 and a concentration of 50 ng/μL, precisely 2 μL of each individual sample to be analyzed was placed in a well of a 384-well plate, together with 2 μL of OpenArray™ Master Mix, using a distribution pattern that obeyed the manufacturer's instructions. The plate was sealed and then given a spin at 1000 rpm for 10 seconds to remove any bubbles present in the mixture.

### Genotyping using the TaqMan® OpenArray™ Genotyping Platform and validation

**Figure 2:**
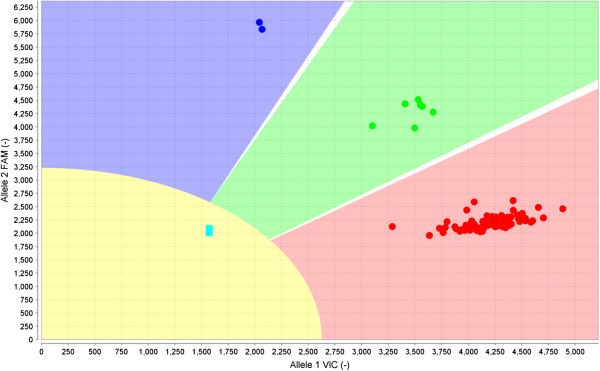
**View of mutation assay p.M34T using the OpenArray™ plate and TaqMan® Genotyper software.** The red cluster represents samples with the normal sequence for both alleles. The green cluster includes individuals having one normal allele and one mutant allele (heterozygous). The blue cluster represents homozygous mutants containing both copies of the mutant alleles. The yellow region with the two blue squares corresponds to the fluorescence of the controls (NTC).

The samples, tips, and the OpenArray™ plate were placed into the AccuFill® system, and the plates were customized to provide an even distribution. A volume of 33 nL was dispensed into each nano-well. After the plates were stabilized with the immersion fluid in a glass case (both items were provided in the kit accompanying the plates), and then sealed with a photosensitive gum for 2 minutes under ultraviolet light in the sealing station. The reaction for the amplification of fragments was performed in the Dual Flat Block GeneAmp® PCR System 9700 thermal cycler (Applied Biosystems), and the cycling lasted approximately 4 hours. The plates were placed in an OpenArray™ NT Cycler® for detection of the emitted fluorescence. After capture of the signal, the results were imported into TaqMan® Genotyper software for interpretation of the results according to the graphs of clusters generated (Figure 2).

The results were tabulated and were then validated using techniques that had been previously established in the same laboratory. The standards listed in Table 3 were used for validation of the tests.

**Table 3:** Techniques used for validation of the assays tested using the OpenArray™ plate

**Gene**	**Mutations analyzed**	**Techniques used to validate**
***GJB2***	p.N206S; p.E147K; p.W24X, c.167delT; c.235delC; p.V95M; p.S113R; p.M93I; p.E129K; p.V37I; p.L90P; p.R184P; p.W172X; p.R75Q; p.M34T; p.V153I; p.K168R; c.35delG; p.R184W	Direct sequencing
***GJB6***	c.6013G > T	Multiplex PCR
***miR96/183***	c.13G > A; c.14C > A	Direct sequencing
***MT-RNR1***	m.1555A > G	*RFLP-PCR*
	m.1494C > T	*RFLP-PCR*
***MT-TS1***	m.7445A > G	*RFLP-PCR*
***SLC26A4***	p.G149R; p.Q413R; p.V609G	Direct sequencing
***OTOF***	p.Q829X	*RFLP-PCR*
***TMC1***	p.S647P	Direct sequencing
***CRYL1***	c.1622 T > C	Direct sequencing

## Results

After selecting the OpenArray™ plate layout, a study was performed using the 32 main mutations associated with hearing loss in the Brazilian population (Table 2). The test for mutation m.827A > G, present in the *MT-RNR1* gene, showed problems during the customization procedure; the manufacturer had not added it to the OpenArray™ plate required for the study, so as a result there were only 31 assays available for each OpenArray™ plate.

In total, 376 samples were studied, obtained from 282 individuals with hearing loss and 94 normal controls. All samples were analyzed in duplicate, searching for the presence of previously selected alterations. Of the total, 367 samples had a good level of purity (A_260/280_ between 1.70 and 2.00) and a concentration of 50 ng/μL.

The number of samples studied was equivalent to four OpenArray™ plates with the layout to analyze 32 assays for 96 individuals (94 patients and two blank controls). In total, 8 plates were used because all the analyses were performed in duplicate to determine whether there were any differences between the results obtained (no differences were observed).

For each OpenArray™ plate, 2,914 genotyping reactions were performed, totaling 11,656 reactions in duplicate to perform the genotyping of the 376 samples. Only nine samples could not be analyzed (279 reactions, equivalent to 2.39% of the total). A further 33 reactions (0.28%) showed different results when compared with the previous results. The tests that were either considered invalid or lacked amplification of the fragments for probe pairing corresponded to 39 reactions (0.33%).

Excluding the failed reactions and divergent results (351 assays, equivalent to 3.01% of the total number of assays), the reproducibility and accuracy were obtained for 96.99% of the reactions (equivalent to 11,305 reactions). Studies of sensitivity and specificity of the technique were performed having 0.97 and 0.98 as average values of these parameters, respectively.

The use of the TaqMan® OpenArray™ Genotyping Platform helped in the genotyping of the selected mutations in 196 samples (52.13% of the samples analyzed) for whom there were no previously established genetic results.

Among the 31 mutations analyzed in 376 samples, 179 changes were detected using the TaqMan® OpenArray™ Genotyping Platform. The results were validated for only 159 alterations, so that there was a discrepancy of 20 changes, classified as false positives and negatives (Table 4).

**Table 4:** Changes detected on the OpenArray™ plates analyzed, and the mutations detected during the validation

**Gene**	**Alterations**	**OpenArray™ cases**	**Validated cases**
***GJB2***	c.35delG/N	40	34
	c.35delG/ c.35delG	34	31
	p.M34T/N	14	11
	p.M34T/ p.M34T	2	2
	p.K168R/N	6	6
	p.V37I/N	4	3
	p.L90P/N	6	6
	c.167delT/N	3	3
	p.R143W/N	2	2
	p.V153I/ p.V153I	1	1
	p.V153I/N	2	1
	p.E147K/N	2	2
	p.R184P/N	4	4
	p.R184W/N	2	2
	p.E129K/N	2	1
	p.W172X/ p.W172X	1	1
	p.N206S/N	2	2
	p.V95M/N	0	1
	p.W24X/N	3	2
	c.35delG/N + p.V95M/N	3	3
	c.35delG/N + p.V37I/N	2	2
	c.35delG/N + p.R184P/N	2	2
	c.35delG/N + p.W24X/N	2	2
	c.35delG/N + c.167delT/N	2	2
	c.35delG/N + p.V153I/N	1	1
	c.35delG/N + p.L90P/N	1	1
	p.V37I/N + p.V95M/N	2	2
	p.V37I/N + c.167delT/N	1	1
	p.M34T/N + p.N206S/N	1	1
	p.M34T/N + p.R184W/N	1	1
	p.V609G/N + p.E129K/N	1	1
	p.V609G/N + p.K168R/N	1	1
***GJB6***	del(GJB6-D13S1830)/N	0	3
	del(GJB6-D13S1854)/N	0	1
***MT-RNR1***	m.A1555G	6	6
***MT-TS1***	m.A7445G	1	1
***SLC26A4***	p.V609G/ p.V609G	3	1
	p.V609G/N	16	10
	p.G149R/N	3	1
	p.Q413R/N	0	1

The cost of genotyping using the TaqMan® OpenArray™ Genotyping Platform for a layout of 32 assays and 96 samples was $600 per plate. Given that it was possible to perform 2,914 reactions per plate, the cost of each reaction would then be approximately $0.20. Therefore, genotyping performed using the selected layout would cost $6.40 per individual analyzed. In all, mutations in 9 different genes were analyzed using this genotyping platform. The values cited above refer only to the kits used for genotyping. Other expenses, such as the value of the equipment, skilled labor, and other reagents were not included in the calculations.

## Discussion

The OpenArray™ genotyping plates showed high performance, with the genotyping call average success rate of 98.85%. This rate represents the amount of genotyping performed with success, in percentage, for OpenArray™ plate. But this value refers to the raw results, including the false positives and negatives. After exclusion of these tests that gave incorrect genotypes, the accuracy rate of the selected layout was 96.99%.

The failed reactions could have been due to samples that were degraded, less pure than recommended (A_260/280_ below 1.7), presented concentrations lower than 50 ng/μL, do not respecting the minimum number of 250 copies of haploid DNA required for the experiment.

False positives were detected in eight assays (Table 4), as follows: c.35delG, p.M34T, p.V37I, p.V153I, p.E129K, and p.W24X in the *GJB2* gene, and p.V609G and p.G149R in the *SLC26A4* gene. All the tests mentioned above were validated by direct sequencing, using an established standard technique for analysis of the *GJB2* and *SLC26A4* genes at the Human Molecular Genetics Laboratory of CBMEG/UNICAMP. These assays were not excluded from the selected layout because some of them are essential. Savings in terms of time and cost could be achieved by only sequencing those samples that showed mutations using the OpenArray™ plates, instead of sequencing all patients in order to detect changes, discarding the false positives.

The tests that showed false negatives concerned p.V95M in the *GJB2* gene and p.Q413R in the *SLC26A4* gene. Heterozygosis samples for these assays were not detected using the OpenArray™ plate, but were confirmed in the validation. However, two samples showed compound heterozygous mutations for the changes p.V37I and p.V95M, and the results were validated by direct sequencing. This last result did not exclude the possibility of a false negative, so the test should be either redesigned or deleted from the chosen layout and replaced by another. In the case of the p.Q413R change, even the positive control for the mutation was not detected. This control was clustered together with the normal subjects, indicating that the test was unable to detect this change. It would be necessary to consider altering the probes and primers in this assay in order to optimize the screening of this alteration. If unsuccessful, it should be deleted from the selected layout and replaced by another assay, chosen following the same criteria used to select the other tests.

The tests rs11843171 (c.6013G > T in the *GJB6* gene) and rs144457142 (c.1622 T > C in the *CRYL1* gene) was selected to try to detect the large *GJB6* gene deletions, but they were uninformative. Both SNPs (Single Nucleotide Polymorphisms) were selected to detect the del(*GJB6*-D13S1830) and/or del(*GJB6*-D13S1854) deletions in the *GJB6* gene. However, these tests failed to detect the deletion of four selected cases (three cases of del(*GJB6*-D13S1830) and one of del(*GJB6*-D13S1854)), which could therefore be considered to be false negatives. However, these SNPs were considered uninformative since the only conclusion that could be reached using this assay would be the presence of a heterozygous sample for one of the SNPs, indicating that the two different probes were paired where there could be deletions. Hence, the individual would be deemed normal, showing no large deletions in the *GJB6* gene.

As reported in the literature, hearing loss is clinically and genetically heterogeneous [37]. In this study, a prevalence of c.35delG cases was detected, present as heterozygous and homozygous forms or in combination with other changes related to hearing loss (compound heterozygous or homozygous). Given such heterogeneity, the search for tools and technologies to perform the screening of major mutations related to hearing loss in the population is intensifying [15]. High-throughput platforms are the main solution for simultaneous screening, as they are much faster compared to conventional molecular techniques used to scan for large numbers of changes (ranging from one change to thousands of changes).

Aspects of the OpenArray™ technology that make it an excellent tool for use in molecular diagnosis is that the plates already contain the assays in the nano-wells, only requiring the addition of the properly quantified DNA sample. Handling and distribution of the samples in the OpenArray™ plate is performed by an auto-pipettor, which increases the precision and decreases pipetting errors. Furthermore, depending on the workflow layout, it is possible to achieve up to around 99,000 genotyping reactions per day. In practice, this scale of analysis is not recommended, due to risks such as evaporation and exchange of samples. Another important point in relation to cost is that compared to conventional genotyping techniques (where tests are performed one at a time), use of the platform is less expensive in terms of both reagents and manual labor.

The use of the high-throughput TaqMan® OpenArray™ Genotyping platform for diagnosis would greatly assist in the initial screening of hearing loss of genetic origin, since it enables the main changes to be evaluated, generating a greater number of results (simultaneously) in a much shorter time compared to the same screening performed using conventional techniques. It is also less expensive. An additional consideration is that after optimization and standardization, the accuracy of the technique (around 97%) would tend to increase due to exchange of the tests that did not work correctly previously, hence optimizing the assays. Due to the genetic heterogeneity of hearing loss, the ideal would be to use a layout with a larger number of Assays (256 Assays for 12 patients), but the basic cost of this layout would increase. Nevertheless, the technique still remains advantageous because the simultaneous screening of 256 alterations can provide a greater number of outputs than conventional techniques, with savings in terms of time and cost.

## Conclusions

The TaqMan® OpenArray™ Genotyping Platform proved to be highly effective for the genotyping of samples in relation to hearing loss, with an accuracy rate of 96.99%, equivalent to 11,305 reactions. The time required to perform the screening of 31 mutations was much lower compared to detection by conventional techniques. Furthermore, the OpenArray™ plates proved to be excellent tools for reproducible diagnosis, since it was only necessary to add the samples to the plate. However, the correct choice and standardization of assays was necessary.

The chosen layout presented two false negative assays (p.V95M in the *GJB2* gene and p.Q413R in the *SLC26A4* gene), which were eliminated using a diagnostic procedure. Eight false positive tests (c.35delG, p.M34T, p.V37I, p.V153I, p.E129K, and p.W24X in the *GJB2* gene, and p.V609G and p.G149R in the *SLC26A4* gene) were not discarded because they are essential tests for the screening.

The rs11843171 (c.6013G > T in the *GJB6* gene) and rs144457142 (c.1622 T > C in the *CRYL1* gene) tests selected to study the del(*GJB6*-D13S1830) and del(*GJB6*-D13S1854) deletions in the *GJB6* gene were found to be uninformative. However, no individuals heterozygous for rs11843171 and rs144457142 were detected using the OpenArray™ platform.

Analysis of the failed results showed that the high yield and reproducibility of this technique were closely associated with the integrity of intact bands in the agarose gel, purity (A_260/280_ between 1.7 and 2.0) and the sample concentration (50 ng/μL).

Although it has been shown to be a promising technique, it has some important limitations to be described. Among the most important are the inability to customize some assays according to the predetermined standards by the company, as the case studies involving insertions and deletions where it is possible to analyze small *indels* of up to 6 bp, and the impossibility of finding new alterations, once the OpenArray™ assays are mounted only with changes already reported or with the known sequence. However, among the strengths of the technique that made it so promising, are the low cost and reduced time to carry out a large number of genotyping, the high accuracy rate, sensitivity, specificity and the easy reproducibility compared to conventional techniques of genotyping. All these points make the platform layout suitable for possible applications involving the diagnosis of not only hearing loss, but also any disease that has known genetic causes.

## Competing interests

The authors declare that they have no competing interests.

## Authors’ contributions

FTAM realized all OpenArray™ experiments, since the samples preparation (evaluation of purity, quantification, dilutions) until the genotyping using the OpenArray™ plates and the validation. PZR and MCCMS helped in the sample preparations, in the plate’s validation and in the manuscript review. AMC and ELS were important in the comments and written of the manuscript. All authors read and accepted the final version of the manuscript.

## Pre-publication history

The pre-publication history for this paper can be accessed here:

http://www.biomedcentral.com/1471-2350/14/112/prepub
